# Living-Donor Liver Transplantation for Hepatocellular Carcinoma: Impact of the MELD Score and Predictive Value of NLR on Survival

**DOI:** 10.3390/curroncol29060310

**Published:** 2022-05-29

**Authors:** Hao-Chien Hung, Jin-Chiao Lee, Yu-Chao Wang, Chih-Hsien Cheng, Tsung-Han Wu, Ting-Jung Wu, Hong-Shiue Chou, Kun-Ming Chan, Wei-Chen Lee, Chen-Fang Lee

**Affiliations:** 1 Department of Liver and Transplantation Surgery, Chang-Gung Memorial Hospital at Linkou, 5 Fusing St., Gueishan Dist., Taoyuan City 333, Taiwan; mp0616@cgmh.org.tw (H.-C.H.); b9302012@cgmh.org.tw (J.-C.L.); b9002072@cgmh.org.tw (Y.-C.W.); chengcchj@cgmh.org.tw (C.-H.C.); domani@cgmh.org.tw (T.-H.W.); wutj5056@cgmh.org.tw (T.-J.W.); chouhs@cgmh.org.tw (H.-S.C.); chankunming@cgmh.org.tw (K.-M.C.); weichen@cgmh.org.tw (W.-C.L.); 2 College of Medicine, Chang-Gung University, Taoyuan City 333, Taiwan

**Keywords:** liver cancer, liver failure, predictive factors, posttransplant outcomes, living-donor liver transplantation

## Abstract

Background: Patients with hepatocellular carcinoma (HCC) tend to be referred for liver transplantation (LT) at an early stage of cirrhosis, with lower pre-LT Model of End-Stage Liver Disease (MELD) scores. We investigated the impact of high MELD scores on post-LT outcomes in patients with HCC and validated the prognostic significance of the neutrophil-to-lymphocyte ratio (NLR). Patients and Method: This retrospective single-center cohort study enrolled 230 patients with HCC who underwent LDLT from 2004–2019 in our institute. We defined a high MELD score as ≥20. Results: The MELD < 20 and MELD ≥ 20 groups comprised 205 and 25 cases, respectively. Although there was no significant difference in disease-free survival between the two groups (*p* = 0.629), the incidence of septic shock (*p* = 0.019) was significantly higher in the high MELD group. The one-, three-, and five-year overall survival rates were not significantly different between the two groups (*p* = 0.056). In univariate analysis, a high pre-LT NLR was associated with poorer survival in the high MELD group (*p* = 0.029, hazard ratio [HR]: 1.07, 90% confidence interval [CI]: 1.02–1.13). NLR cut-off values of ≥10.7 and <10.7 were predictive of mortality, with an AUC of 0.705 (90% CI: 0.532–0.879). The one-, three-, and five-year post-LT survival rates were significantly higher among the recipients with an NLR < 10.7 than those with an NLR ≥ 10.7 (*p* = 0.005). Conclusions: Pre-LT MELD score ≥ 20 was associated with a higher risk of developing post-LT septic shock and mortality. The pre-LT serum NLR is a useful predictive factor for clinical outcomes in patients with HCC with high MELD scores.

## 1. Introduction

Since the introduction of Milan’s criteria [1], the long-term survival rates after liver transplantation (LT) for patients with hepatocellular carcinoma (HCC) have improved significantly. Thereafter, a variety of expanded criteria have been proposed, and recipients with HCC who meet these conditions have shown comparable outcomes to patients without HCC with advanced cirrhosis or liver failure [1,2,3,4]. To avoid dropout from the waiting list due to tumor progression, patients with HCC can be referred for transplantation when their native livers are in the early stage of cirrhosis or not yet cirrhotic. As a result, recipients with HCC often have a low Model of End-stage Liver Disease (MELD) score at transplant. However, there are limited data on the results of transplant in patients with HCC with high MELD scores.

Although some studies have reported that high MELD scores do not adversely affect patient or graft outcomes [5,6], recipients with a higher MELD score are usually recognized as a high-risk group after LT. Owing to their poorer health, they are more vulnerable to infection and are in a dangerous position to encounter post-LT complications [7,8,9,10], which raises the question of whether this immunocompromised situation causes decreased anti-tumor immunity and higher rates of tumor recurrence in this patient group [5]. Therefore, it is desirable to identify a reliable surrogate marker that is representative of pre-LT immunity in patients with high MELD scores to predict post-LT outcomes, especially in patients with concomitant HCC.

The neutrophil-to-lymphocyte ratio (NLR), a peripheral inflammatory parameter, has been shown to be useful in predicting graft dysfunction, acute rejection, and high-risk explant features [11,12,13,14,15]. It is also widely used as a valid indicator of prognosis in solid tumors [16,17]. Based on the above, we were interested in applying the NLR to our patient group. We aimed to investigate the impact of high MELD scores on the clinical outcomes of patients with HCC who underwent living-donor liver transplantation (LDLT). We also attempted to validate the predictive value of the NLR for adverse prognosis in this high-risk subpopulation.

## 2. Materials and Methods

### 2.1. Study Design, Setting, and Population

This was a retrospective single-center cohort study. We screened 746 adults who underwent LDLT between October 2004 and October 2019 at the Chang Gung Memorial Hospital, Linkou, Taiwan. We excluded patients without HCC or those who had an insufficient follow-up period to monitor the outcome. Ultimately, 230 HCC recipients with different levels of end-stage liver disease were enrolled. We followed the University of California San Francisco (UCSF) criteria (solitary tumor ≤ 6.5 cm or up to three tumors < 4.5 cm) for patient selection [2], which was judged from the latest radiological findings prior to transplantation. The study protocol was approved by the Ethics Committee and the Institutional Review Board of the Chang Gung Memorial Hospital (approval no. 202101491B0).

### 2.2. Liver Transplantation Protocol

The pre-operative preparation and LDLT techniques were executed in accordance with our institutional protocol [18,19]. In general, an estimated graft-to-recipient weight ratio (GRWR) < 0.7% was regarded as high risk, and most of our recipients received the right liver lobe. Biochemical analyses, such as MELD score, Child-Pugh score, and serum NLR, were obtained from blood samples collected within 24 h before transplantation. The NLR was calculated by dividing the absolute number of neutrophils, according to the absolute number of lymphocytes. An NLR value ≥ 5.0 was considered elevated.

Post-LT immunosuppressant administration was introduced in our previous publications [20], in which primary immunosuppression consisted of tacrolimus, corticosteroids, and mycophenolate mofetil (1 g/day). The target trough tacrolimus level was maintained at 6–10 ng/mL. Steroids were tapered off within 3 months after LT. For patients without liver biopsy, the diagnosis of acute rejection was made based on clinical evidence of rapid elevation (>30 IU/L) of serum aspartate aminotransferase (AST) and alanine aminotransferase (ALT) within 24 h, not due to other causes of hepatic transaminase increase.

Alpha-fetoprotein (AFP) and Doppler ultrasound were performed for tumor surveillance every 3 months after LT, and computed tomography was conducted every 6 months or when necessary. HCC recurrence after LT was defined as a tumor found in any location throughout the body following a period when it was undetectable. The disease-free survival (DFS) was calculated from LT to the day of tumor recurrence.

### 2.3. Clinical Outcomes Assessment

The primary outcome was long-term overall survival (OS), which was calculated from the transplantation date to the date of death; data from patients who were alive as of 31 June 2021 were censored. Several types of infection were considered in our study. Post-LT infection, defined as any culture evidence of invasion and growth of pathogens (bacteria, viruses, yeast, fungi, or other microorganisms) in recipients. Detectable cytomegalovirus (CMV) antigen or CMV DNA in the patient’s blood was a prerequisite for CMV disease in the presence of clinical symptoms or signs [21]. Severe CMV disease occurs when CMV disease progresses and causes failure of two or more organ systems [22]. Septic shock is defined as profound sepsis leading to a low systolic blood pressure (<90 mmHg), which requires vasopressors or inotropes during the same hospital stay after LT.

### 2.4. Statistical Analysis

Continuous variables are reported as median values and mean values ± standard deviations, with a range of minimum and maximum ranges, while categorical variables are reported as numbers (percentages). Pearson’s chi-square test, independent t-test, and nonparametric methods were used to compare clinical parameters. The Cox proportional hazard risk model was the choice of time-to-event outcome regarding OS to reveal the effect of each parameter, and a binary logistic regression model was used to calculate the relationship between several risks and dichotomous events (the presence and absence of post-LT septic shock). In the univariate analysis, all potential parameters were included, and only factors with a *p*-value of <0.100 were subsequently entered into the multivariate analysis. The Kaplan–Meier method with a log-rank test was used to compare patient survival between the groups. All statistical analyses were performed using IBM SPSS^®^ (version 24.0; SPSS Inc., Chicago, IL, USA), and two-tailed *p*-values of <0.05 were considered statistically significant. Receiver operating characteristic (ROC) curve analysis was performed to determine the predictive value of a clinical factor, and the optimal cut-off point was determined using the Youden index to assess the predictive accuracy. Linear regression analysis was used to assess the relationship between the MELD score and serum NLR.

## 3. Results

### 3.1. Demographics of the Enrolled Recipients

After reviewing all of the recorded data, the demographic and laboratory characteristics of all 230 patients are summarized below (Table S1). The mean age at which the recipients received LDLT was 55.5 ± 7.2 years (range, 32.8–70.3 years). The majority of the population comprised male patients (*n* = 186, 80.9%) and those with an etiology of viral hepatitis (*n* = 219, 95.2%). The mean Child-Pugh and MELD scores were 7.7 ± 2.2 (range, 5–12) and 12.9 ± 5.7 (range, 6–36), respectively. Among them, 189 (82.2%) patients received locoregional therapy targeting HCC at least once before LT. Two hundred and eighteen (94.8%) recipients obtained a right liver graft, with a mean GRWR of 0.95 ± 0.18% (range, 0.57–1.43%). Explant histology was examined by a certified hepatopathologist, and the findings showed that 63 (27.4%) patients had tumor status beyond the UCSF criteria. The mean size of the maximum tumor and the summation of all tumors were 2.7 ± 1.5 cm (range, 0.5–11.2) and 4.9 ± 3.6 cm (range, 0.5–19.5), respectively. The mean tumor number was 2.7 ± 2.7, and the median for the given data was 2.

### 3.2. Prognostic Factors Affecting Post-LT Outcomes

We investigated independent pre-operative factors to predict post-LT long-term survival (Table S2). Our results showed that pre-operative factors, such as recipient and donor age, recipient and donor sex, viral hepatitis, serum AFP, tumor status, Child-Pugh score, and NLR were not potential predictors in the univariate analysis. Only pre-transplant MELD scores of ≥20 were relevant for survival prediction. Therefore, we defined ≥20 as a high MELD score, which was used as the basis for subsequent grouping. As infection remains the major leading cause of death in the early post-LT period, it is logically justifiable to determine which infectious status is of great significance in terms of affecting survival.

Subsequent multivariate analysis revealed a predominant weight of septic shock in survival prediction (Table S3). Post-LT septic shock had a strong impact on survival (*p* < 0.001).

The leading cause of post-LT infection was a blood-stream infection or catheter-associated infection, followed by similar proportions of intra-abdominal infection and pneumonia. Eighteen and six patients experienced septic shock in the low and the high MELD group, respectively. Besides this, over half of low-MELD patients with septic shock (*n* = 10/18, 55.6%) and 2 high-MELD patients (*n* = 2/6, 33.3%) with septic shock were diagnosed with pneumonia, which seemed to have a great significance in developing this lethal situation.

### 3.3. Comparisons of the Characteristics between the High and the Low MELD Score Groups

A comparison of demographic data between the high (≥20) and low (<20) MELD score groups is summarized in Table 1. The MELD < 20 group comprised 205 (89.1%) recipients, and the MELD ≥ 20 group comprised 25 (10.9%). In the high MELD score group, the mean MELD score was 25.0 ± 5.0, and 11.4 ± 3.7 in the low MELD score group. The high MELD score group had an increased incidence of chronic HCV infection, higher Child-Pugh scores, higher NLR levels before LT, and increased intra-operative ascites and blood loss (all *p*-values < 0.05), confirming that the pre-transplant condition and intra-operative course were more complicated in the high MELD score group. Most oncological factors (AFP, explant tumor characteristics, tumor status by radiologic or pathologic assessment, etc.) were not significantly different between the MELD < 20 and MELD ≥ 20 groups. Notably, there were fewer patients who received locoregional treatment for HCC before LT in the high MELD group (*n* = 14, 56.0% vs. *n* = 175, 85.4%; *p* < 0.001). Details were accessed according to different intentions of locoregional treatment before LT, and there were 11 (5.4%) and 1 (4.0%) for curative intention, 143 (69.8%) and 11 (44.0%) for bridge therapy before LT, 21 (10.2%) and 2 (8.0%) for tumor down-staging in the low and the high MELD group, respectively. Regarding the proportions of those receiving down-staging therapy, there was no significant difference in the two groups (*p* = 0.801). Most of the recipients had TACE-based locoregional therapy, except seven patients in the low MELD group and 1 case in the high MELD group, who received radiofrequency ablation only. Concerning the observation time from the latest locoregional treatment to the time of transplantation, there were 88 of the 175 (50.3%) and 9 of the 14 (64.3%) patients who received LRT with at least a 3 month period in the low and the high MELD group, respectively (*p* = 0.313).

### 3.4. Comparisons of Clinical Outcomes between the High and the Low MELD Score Groups

**Figure 1:**
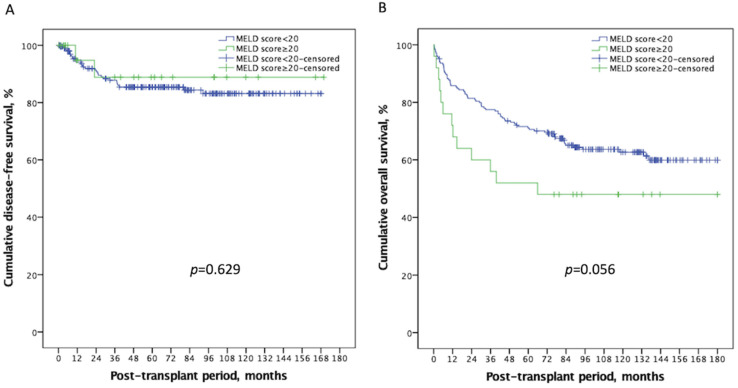
Kaplan–Meier plot of disease-free survival and overall survival according to a MELD score ≥ 20 or <20. (**A**) There was no significant difference in disease-free survival (*p* = 0.629) between the two groups. (**B**) Recipients with a high MELD score ≥ 20 had a worse overall survival than those with a low MELD score < 20 (*p* = 0.056). MELD: Model of end-stage liver disease.

Next, we examined whether high MELD scores had a negative impact on post-transplant outcomes. Tumor recurrence, acute rejection, and in-hospital infections were considered to be associated with post-LT survival and were compared between the two groups. The mean follow-up period of the present study was 83.65 ± 52.80 months (the median value: 87.42 months; range: 0.20–185.52 months). Table 2 summarizes these results and lists the causes of death systematically according to the high and low MELD scores. There was no significant difference in HCC DFS between the two groups. The 1-, 3-, and 5-year DFS were 93.1%, 85.7%, 83.4% for patients in the low MELD group, and 94.7%, 82.9%, 82.9% for patients in the high MELD group (Figure 1A, *p* = 0.629). Over half of the mortalities were infection-related, followed by HCC recurrence. Regarding infections, CMV disease (*p* = 0.007), severe CMV disease (*p* = 0.028), and septic shock (*p* = 0.019) were significantly more prevalent among the high MELD group. As a result, the OS was worse in the high MELD score group; the 1-, 3-, and 5-year OS rates were 85.8%, 77.5%, 71.1% for patients in the low MELD group, and 72.0%, 56.0%, 52.0% for patients in the high MELD group, respectively (Figure 1B, *p* = 0.056). These results support the view that pre-transplant immunity was indeed more compromised in the high MELD group, although this weakened condition did not lead to significant tumor recurrence.

In Table 3, we performed uni- and multi-variate analyses to identify the independent predictors of post-LT septic shock in all enrolled patients with HCC after LDLT. The results showed that pre-operative factors, such as recipient and donor age, recipient and donor sex, viral hepatitis, and serum AFP, were not potential predictors in the univariate analysis, but MELD score ≥ 20, NLR ≥ 5, pre-LT locoregional treatment before LT were. In the multivariate logistic model, only high MELD score had a significant interaction with subsequent septic shock (*p* = 0.025, HR: 3.28, 90% CI: 1.37–7.84). We confirmed that a high MELD score is an independent risk factor for post-LT septic shock, which could lead to poor prognosis.

### 3.5. Impact of NLR Affecting Outcomes in HCC Recipients with MELD Score ≥ 20

We further investigated negative predictors in the high-risk subgroup. Univariate analysis was performed for pre-operative factors to predict septic shock and survival using the Cox regression model in patients with HCC with a high MELD score (≥20) receiving LDLT (Table 4). The following pre-operative factors were calculated in univariate analysis: recipient age, recipient sex, chronic hepatitis B virus infection, chronic hepatitis C virus infection, MELD score, pre-LT serum NLR, pre-LT locoregional treatment, serum AFP level, donor age, and donor sex. The results revealed that a high pretransplant serum NLR not only had a negative influence on post-LT septic shock (*p* = 0.027, HR: 1.13, 90% CI: 1.02–1.27), but also compromised post-LT survival (*p* = 0.029, HR: 1.07, 90% CI: 1.02–1.13).

**Figure 2:**
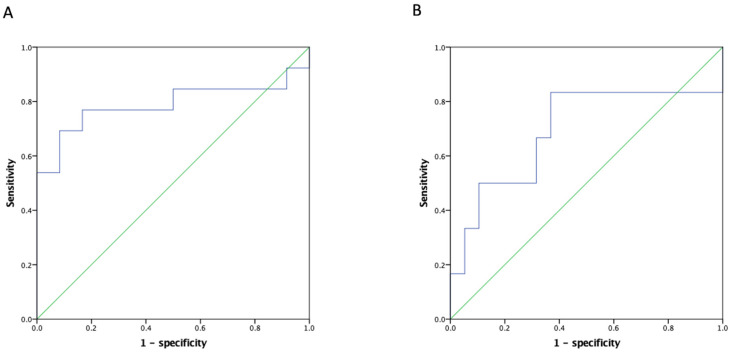
ROC of NLR for HCC recipients with a high MELD score in predicting (**A**) overall survival and (**B**) septic shock after LDLT. The C-index of the NLR in predicting mortality was 0.788 (90% CI: 0.591–0.986) and that of septic shock was 0.693 (90% CI: 0.404–0.982). ROC: Receiver operating characteristic curve, C-index: Concordance index, NLR: Neutrophil-to-lymphocyte ratio, HCC: Hepatocellular carcinoma, MELD: Model of end-stage liver disease, LDLT: Living donor liver transplantation.

**Figure 3:**
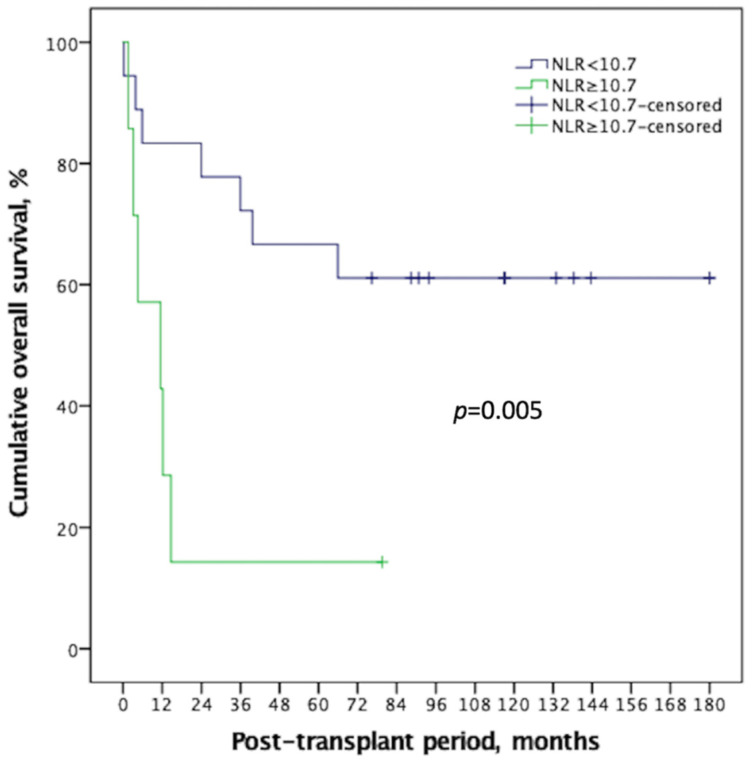
NLR showed good discrimination on Kaplan–Meier survival comparison for HCC recipient with high MELD score, with an ideal cut-off value of 10.7. There was significant survival difference (*p* = 0.005) between the two groups, and the high NLR group demonstrated a long-term survival rate < 20%. NLR: neutrophil-to-lymphocyte ratio, HCC: Hepatocellular carcinoma, MELD: Model of end-stage liver disease.

ROC analyses were performed to quantify pre-transplant serum NLR among recipients with a high MELD score to determine the optimal cut-off thresholds to predict the two main clinical outcomes (14.0 for post-LT septic shock and 10.7; mortality). The Concordance index (C-index) of NLR in predicting mortality was 0.788 (90% CI: 0.591–0.986; Figure 2A), and that of septic shock was 0.693 (90% CI: 0.404–0.982; Figure 2B). Using these optimal divisions, the correlated sensitivity, specificity, positive predictive value, negative predictive value, and accuracy were 50.0%, 84.2%, 50.0%, 84.2%, and 76.0% for NLR divided by 11.0, and 91.7%, 46.2%, 57.2%, 87.6%, and 68.0% for NLR divided by 10.7 in anticipating occurrences of septic shock and post-LT mortality, respectively. The 1-, 3-, and 5-year post-transplant survival rates were 83.3%, 72.2%, and 66.7% among the recipients with an NLR < 10.7, and 42.9%, 14.3%, and 14.3% among those with an NLR ≥ 10.7, respectively (*p* = 0.005; Figure 3).

**Figure 4:**
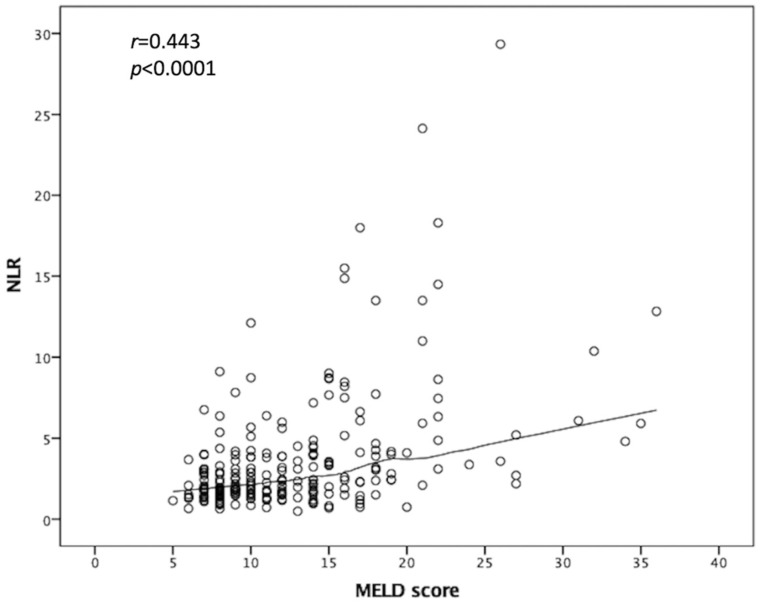
Relationship between MELD score and NLR value. Linear regression was performed, which showed a moderate correlation between MELD score and serum NLR. NLR: Neutrophil-to-lymphocyte ratio, MELD: Model of end-stage liver disease.

As the pre-LT serum NLR demonstrates good discriminative power in predicting both OS and development of septic shock, we were interested in the relationship between the MELD score and NLR values. Consequently, linear regression was performed for the 230 patients, which showed a moderate correlation between the MELD score and serum NLR (r = 0.443, *p* < 0.0001; Figure 4). These data suggest that the NLR is a representative marker of pretransplant immunity and a predictor of post-LT outcomes.

## 4. Discussion

As the demand for liver transplantation for HCC treatment is increasing, identifying patients at a greater risk for LT is needed to improve overall outcomes. Several prognostic factors have been reported to influence survival, including recipient and donor age, HCC, HCV cirrhosis, and operation time [23]. Septic shock after solid organ transplantation is one of the most severe and lethal complications. The development of septic shock is indicative of post-LT mortality [24], and it may be related to old recipient age, nosocomial infections, and pulmonary infectious sources [25,26].

Nevertheless, LT is the treatment of choice for patients with HCC with advanced cirrhosis, and the impact of high pre-LT MELD scores on HCC recipients needs to be elucidated more clearly. Although some researchers regard high pre-LT MELD scores to have no influence on outcomes after LDLT [5,27], many believe that recipients with a high MELD score are less tolerant of marginal graft [28]. Our data emphasized that recipients with a pre-transplant MELD score ≥ 20 had a higher risk of developing post-LT septic shock and mortality, but that the HCC DFS was not significantly different between the high and low MELD score groups. 

The 3-year OS in the current study was 75.1%, which is comparable with other approved centers in Taiwan, with an overall 3-year OS of 82% (ranging 62–91%) [29]. Poor outcomes in high MELD HCC recipients seem to be related to early post-transplant infection. Our results showed that a high NLR was capable of predicting adverse outcomes in HCC recipients with a high MELD score, particularly during the first few months after transplantation. A high serum NLR is expected to affect sepsis-related complications more directly, which are consequences of compromised host immunity or progression from pre-existing subclinical infection. Looking forward to the future, it may be a potential serial biomarker to evaluate recipients with pre-existing infection before LT.

Interactions between NLR and post-LT outcomes in patients are likely to be complex. A high NLR represents a combination of neutrophilia and lymphocytopenia, and the former may indicate an active or unsolved infection, while the latter may imply insufficient host immunity and anti-cancerous ability [30,31,32]. Moreover, deficiency of innate or adaptive immunity could elicit opportunistic infections, and a prolonged post-LT recovery course may also lead to the development of other nosocomial infections. The prognostic value of the NLR has been verified in patients with sepsis [33]. The majority of studies have found that the cut-off value of NLR is approximately 3.0 (interquartile range, 2.5–5.0) [17]. In our study, the optimal cut-off values of the pre-LDLT serum NLR in HCC recipients with a high MELD score to predict post-LT septic shock and OS were 11.0 and 10.7, respectively. These values represent moderate to severe inflammation and stress with high intensity on the NLR meter [17], whether it is cancer-related or not.

Despite a moderate correlation between the MELD score and serum NLR, which has been engaged in a predictive capacity for HCC recurrence [34], our study did not reveal a direct connection between the MELD score and oncologic outcomes. In our cohort, viral hepatitis (especially hepatitis C virus) was significantly more prevalent in recipients with a high MELD score. Previous studies have considered viral hepatitis-associated cirrhosis as a survival risk [35,36]. HBV- and HCV-associated HCC have distinct clinical and pathological characteristics; in contrast to chronic HBV infection, patients with HCC and chronic HCV infection tend to be more affected by advanced cirrhosis [37]. In addition, our patients with high MELD scores received less pre-LT locoregional treatment owing to more severe hepatic dysfunction, including chemo-embolization and ablation. Patients with high MELD scores more urgently require treatment for liver failure than for cancer. These differences will probably necessitate different screening and treatment policies to optimize HCC surveillance and management after transplantation.

This study has several limitations in addition to the retrospective design. First, a type II error may exist because of the relatively small sample size. Second, this research was conducted based on laboratory information at a single time point before LT surgery, rather than the average from peri-operative time frames, and as such, a lack of dynamic evaluation and bias may arise. Third, direct evaluation of independent pre-operative risk factors for survival is beyond the preset accessibility. We focused on septic shock and its impact on post-transplant outcomes. Therefore, larger prospective studies are needed to validate our findings. 

## 5. Conclusions

In conclusion, managing high MELD scores in recipients with HCC remains a challenge. Although the DFS is not worse in these patients, they are more vulnerable to severe infections and develop poorer outcomes. A high pre-LT NLR can be used as a negative predictor of clinical outcomes in this high-risk subgroup. Early recognition of high-risk patients and implementation of prophylactic management are mandatory to prevent post-LT complications and optimize the overall outcomes after LDLT.

## Figures and Tables

**Table 1:** A comparative study of LDLT for HCC patients according to high and low MELD scores.

General Information of Recipients	MELD < 20, *n* = 205	MELD ≥ 20, *n* = 25	*p*-Value
Recipient age, year-old	55.3 ± 7.4	56.8 ± 6.3	0.312
Recipient age ≥ 60-year-old	59 (28.8%)	10 (40.0%)	0.248
Recipient sex, male	169 (82.4%)	17 (68.0%)	0.083
Viral hepatitis			0.018
None	7 (3.4%)	4 (16.0%)	
Chronic HBV infection	138 (67.3%)	11 (44.0%)	
Chronic HCV infection	49 (23.9%)	8 (32.0%)	
Co-infection of HBV and HCV	11 (5.4%)	2 (8.0%)	
Child-Pugh score	7.3 ± 2.1	10.4 ± 1.2	<0.001
MELD score	11.4 ± 3.7	25.0 ± 5.0	<0.001
NLR	3.3 ± 4.0	8.4 ± 7.1	0.001
NLR ≥ 5	29 (14.1%)	15 (60.0%)	<0.001
Locoregional treatment before LT Curative-intent Bridge therapy Down-staging therapy	175 (85.4%) 11 (5.4%) 143 (69.8%) 21 (10.2%)	14 (56.0%) 1 (4.0%) 11 (44.0%) 2 (8.0%)	<0.001
Beyond UCSF criteria, by radiology	16 (7.8%)	3 (12.0%)	0.472
Beyond UCSF criteria, by pathology	56 (27.3%)	7 (28.0%)	0.942
AFP, ng/dL	13.0	9.9	0.672
AFP ≥ 200 ng/mL	29 (14.1%)	1 (4.0%)	0.155
Recipient CMV IgG, positive	191 (93.2%)	24 (96.0%)	0.588
Donor and Operative factors			
Donor age, year-old	30.5 ± 8.4	30.5 ± 9.5	0.968
Donor age ≥ 45-year-old	12 (5.9%)	2 (8.0%)	0.672
Donor sex, male	116 (56.6%)	18 (72.0%)	0.140
Graft type, right liver	193 (94.1%)	25 (100.0%)	0.214
Ascites amount, mL, intraoperative	1200	2500	<0.001
Blood loss, mL, intraoperative	50	1600	0.011

Abbreviation: LDLT, living donor liver transplantation; HCC, hepatocellular carcinoma; MELD, model of end-stage liver disease; HBV, hepatitis B virus; HCV, hepatitis C virus; NLR, neutrophil-to-lymphocyte ratio; UCSF, university of California San Francisco; AFP, alpha-fetoprotein; CMV IgG, cytomegalovirus Immunoglobulin G; GRWR, graft-to-recipient weight ratio.

**Table 2:** Clinical outcomes of LDLT for HCC patients according to high and low MELD scores.

Clinical Outcomes	MELD < 20, *n* = 205	MELD ≥ 20, *n* = 25	*p*-Value
Post-LT HCC DFS rate, at 1-, 3-, 5-year, %	93.1, 85.7, 83.4%	94.7, 82.9, 82.9%	0.973
Post-LT HCC recurrence, number	33 (16.1%)	3 (12.0%)	0.594
Post-LT HCC recurrence, months	23.4 ± 21.7	20.5 ± 8.7	0.825
Acute rejection	55 (26.8%)	7 (28.0%)	0.900
Post-LT infection Urine tract infection Intraabdominal infection Pneumonia Blood stream or catheter related infection	95 (46.3%) 8 (3.9%) 29 (14.1%) 28 (13.7%) 30 (14.6%)	15 (60.0%) 1 (4.0%) 3 (12.0%) 3 (12.0%) 8 (32.0%)	0.197
CMV disease	30 (14.6%)	9 (36.0%)	0.007
Severe CMV disease	10 (4.9%)	4 (16.0%)	0.028
Septic shock	18 (8.8%)	6 (24.0%)	0.019
Major post-LT complication	20 (9.8%)	8 (32.0%)	0.001
Post-LT OS rate, at 1-, 3-, 5-year, %	85.8, 77.5, 71.1%	72.0, 56.0, 52.0%	0.056
Cumulative mortalities Infection-related	76 (37.1%) 41 (53.9%)	13 (52.0%) 7 (53.8%)	0.148
HCC-related	18 (23.7%)	1 (7.7%)	
Rejection-related	8 (10.5%)	3 (23.1%)	
Cardiovascular disease	3 (3.9%)	1 (7.7%)	
Bleeding	2 (2.6%)	1 (7.7%)	
Others	4 (5.3%)	0 (0.0%)	

Abbreviation: LDLT, living donor liver transplantation; HCC, hepatocellular carcinoma; MELD, model of end-stage liver disease; DFS, disease free survival; CMV, cytomegalovirus; OS, overall survival.

**Table 3:** Uni-/multivariate analyses for pre-operative factors to predict post-LT septic shock in all enrolled HCC patients after LDLT using binary logistic regression model by backward selection (likelihood ratio).

Parameters	Univariate			Multivariate		
	HR	90%CI	*p*-Value	HR	90%CI	*p*-Value
MELD score ≥ 20	3.28	1.37–7.84	0.025	3.28	1.37–7.84	0.025
NLR ≥ 5	2.36	1.09–5.12	0.068			
Pre-LT locoregional treatment	0.44	0.21–0.93	0.071			

Abbreviation: HCC, hepatocellular carcinoma; LDLT, living donor liver transplantation; HR, hazard ratio; CI, confidence interval; MELD, model of end-stage liver disease; NLR, neutrophil-to-lymphocyte ratio. Following pre-operative factors (categorized by cut-off values) were calculated in the univariate analysis: Recipient age (60-year-old), recipient sex, viral hepatitis, MELD score (20), pre-LT serum NLR (5), pre-LT locoregional treatment, serum AFP (200 ng/dL), donor age (45-year-old), donor sex; only significant results (*p*-value < 0.100) were shown in this table and entering the multivariate analysis.

**Table 4:** Univariate analyses for pre-operative factors to predict septic shock and survival using cox regression model in HCC patients with high MELD score receiving LDLT.

Predicted Event	Post-LT Septic Shock			Post-LT Mortality		
	HR	90%CI	*p*-Value	HR	90%CI	*p*-Value
NLR	1.13	1.02–1.27	0.027	1.07	1.02–1.13	0.029

Abbreviation: HCC, hepatocellular carcinoma; MELD, model of end-stage liver disease; LDLT, living donor liver transplantation; HR, hazard ratio; CI, confidence interval; NLR, neutrophil-to-lymphocyte ratio. Following pre-operative factors were calculated in the univariate analysis: Recipient age, recipient sex, chronic hepatitis B virus infection, chronic hepatitis C virus infection, MELD score, pre-LT serum NLR, pre-LT locoregional treatment, serum alpha-fetoprotein, donor age, donor sex.

## Data Availability

The data presented in this study are available on request from the corresponding author.

## Supplementary Materials

The following supporting information can be downloaded at: https://www.mdpi.com/article/10.3390/curroncol29060310/s1, Table S1: Characteristics of enrolled 230 HCC patients received LDLT; Table S2: Univariate analyses for pre-operative factors to predict survival in HCC patients after LDLT using cox regression model; Table S3: Uni-/multivariate analyses for different infectious status to predict overall survival in HCC patients after LDLT using cox regression model by backward selection (likelihood ratio).

## Abbreviations

ALT	alanine aminotransferase
AST	aspartate aminotransferase
AUROC	area under the receiver operating characteristic curve
CMV	cytomegalovirus
DFS	disease-free survival
GRWR	graft-to-recipient weight ratio
HCC	hepatocellular carcinoma
LDLT	living-donor liver transplantation
LT	liver transplantation
MELD	Model of End-Stage Liver Disease
NLR	neutrophil-to-lymphocyte ratio
OS	overall survival
ROC	receiver operating characteristic
UCSF	University of California San Francisco
