# Supplementary material for: Living-Donor Liver Transplantation for Hepatocellular Carcinoma: Impact of the MELD Score and Predictive Value of NLR on Survival
Source: Curr Oncol. 2022 May 29;29(6):3881–93. doi: 10.3390/curroncol29060310 (PMC9221955; doi:10.3390/curroncol29060310)
Supplement: Supplementary file 1 [file curroncol-29-00310-s001.zip › curroncol-1721523-supplementary.pdf]

Supplemental table S1. Characteristics of enrolled 230 HCC patients received LDLT

General information	Mean±SD	Number (%) or median	Minimun-maximum
Recipient age, year-old	55.5±7.2	55.9	32.8-70.3
Sex, male		186 (80.9%)	
Viral hepatitis		219 (95.2%)	
Child-Pugh score	7.7±2.2	8	5-12
MELD score	12.9±5.7	12	6-36
NLR	3.9±4.7	2.4	0.5-29.2
Locoregional treatment before LT		189 (82.2%)	
Beyond UCSF criteria, radiology		19 (8.3%)	
AFP, ng/dL, at LT	136.7±361.2	13.7	1.0-2525.0
Recipient CMV IgG, positive		215 (93.5%)	
Living donor liver transplantation			
Donor age, year-old	30.5±8.5	29.1	18.0-58.1
Donor sex, male		134 (58.3%)	
Graft type, right liver		218 (94.8%)	
GRWR, %	0.95±0.18	0.94	0.57-1.43
Ascites amount, mL, intraoperative	1137.3±2463.9	0	0-13800
Blood loss, mL, intraoperative	2041.3±2582.9	1375	50-23400
Explant pathology			
Tumor size, cm (maximum)	2.7±1.5	2.4	0.5-11.2
Tumor size, cm (summation)	4.9±3.6	3.6	0.5-19.5
Tumor number	2.7±2.7	2	1-20
Satellite nodule		20 (8.7%)	
Microvascular invasion		40 (17.4%)	
Tumor capsulation		164 (71.3%)	
Capsule invasion		100 (43.5%)	

Histology grade, ≥ III	54 (23.5%)
Beyond UCSF criteria, pathology	63 (27.4%)

Abbreviation: HCC, hepatocellular carcinoma; LDLT, living donor liver transplantation; SD, standard deviation; HBV, hepatitis B virus; HCV, hepatitis C virus; MELD, model of end-stage liver disease; NLR, neutrophil-to-lymphocyte ratio; UCSF, university of California San Francisco; AFP, alpha-fetoprotein; CMV IgG, cytomegalovirus Immunoglobulin G; GRWR, graft-to-recipient weight ratio.

Supplemental table S2. Univariate analyses for pre-operative factors to predict survival in HCC patients after LDLT using cox regression model

Parameters	HR	90%CI	*p*-value
Recipient age, year-old	0.99	0.97-1.02	0.545
Recipient age ≥ 60-year-old	1.16	0.80-1.69	0.504
Sex, male	0.80	0.52-1.21-	0.366
HBV	1.02	0.70-1.50	0.923
HCV	1.13	0.78-1.64	0.602
Child-Pugh score	1.07	0.96-1.19	0.316
Child-Pugh class, C	1.34	0.79-2.28	0.368
MELD score	1.03	0.99-1.06	0.116
MELD score ≥ 20	1.76	1.08-2.89	0.059
NLR	1.02	0.99-1.05	0.219
NLR ≥ 5	1.47	0.97-2.24	0.129
Locoregional treatment before LT	0.74	0.50-1.11	0.220
Beyond UCSF criteria, radiology	1.25	0.68-2.30	0.545
AFP, ng/dL	1.00	0.99-1.01	0.832
AFP ≥ 200 ng/mL	1.15	0.55-2.40	0.753
Recipient CMV IgG, positive	0.327	0.10-1.07	0.120
Donor/graft information			
Donor age, year-old	1.01	0.99-1.03	0.511
Donor age ≥ 45-year-old	0.525	0.20-1.38	0.273
Donor Sex, male	0.864	0.61-1.23	0.496
Graft type, right liver	0.680	0.34-1.36	0.362

Abbreviation: HCC, hepatocellular carcinoma; LDLT, living donor liver transplantation; HR, hazard ratio; CI, confidence interval; HBV, hepatitis B virus; HCV, hepatitis C virus; MELD, model of end-stage liver disease; NLR, neutrophil-to-lymphocyte ratio; UCSF, university of California San Francisco;

AFP, alpha-fetoprotein; CMV IgG, cytomegalovirus Immunoglobulin G; GRWR, graft-to-recipient weight ratio.

Supplemental table S3. Uni-/multivariate analyses for different infectious status to predict overall survival in HCC patients after LDLT using cox regression model by backward selection (likelihood ratio)

	Univariate			Multivariate		
	HR	90%CI	*p*-value	HR	90%CI	*p*-value
CMV disease	2.56	1.61-4.07	<0.001			
Severe CMV infection	6.39	3.51-11.64-	<0.001			
Post-LT infection	2.05	1.38-3.15	0.001	1.52	0.94-2.47	0.088
Septic shock	7.57	4.62-12.40	<0.001	5.80	3.28-10.26	<0.001

Abbreviation: HCC, hepatocellular carcinoma; LDLT, living donor liver transplantation; HR, hazard ratio; CI, confidence interval; CMV, cytomegalovirus.
